# Wheat root systems as a breeding target for climate resilience

**DOI:** 10.1007/s00122-021-03819-w

**Published:** 2021-04-26

**Authors:** Eric S. Ober, Samir Alahmad, James Cockram, Cristian Forestan, Lee T. Hickey, Josefine Kant, Marco Maccaferri, Emily Marr, Matthew Milner, Francisco Pinto, Charlotte Rambla, Matthew Reynolds, Silvio Salvi, Giuseppe Sciara, Rod J. Snowdon, Pauline Thomelin, Roberto Tuberosa, Cristobal Uauy, Kai P. Voss-Fels, Emma Wallington, Michelle Watt

**Affiliations:** 1 NIAB, 93 Lawrence Weaver Road, Cambridge, CB3 0LE UK; 2 Centre for Crop Science, Queensland Alliance for Agriculture and Food Innovation, The University of Queensland, Brisbane, QLD 4072 Australia; 3 Department of Agricultural and Food Sciences, University of Bologna, Viale G Fanin 44, 40127 Bologna, Italy; 4 Forschungszentrum Jülich, IBG-2, Wilhelm-Johnen-Straße, 52428 Jülich, Germany; 5 Global Wheat Program, International Maize and Wheat Improvement Center (CIMMYT), 56237 Texcoco, Estado de Mexico Mexico; 6 Department of Plant Breeding, IFZ Research Centre for Biosystems, Land Use and Nutrition, Justus Liebig University, Heinrich-Buff-Ring 26-32, 35392 Giessen, Germany; 7 John Innes Centre, Norwich Research Park, Colney Lane, Norwich, NR4 7UH UK; 8 Centre for Animal Science, Queensland Alliance for Agriculture and Food Innovation, The University of Queensland, Brisbane, QLD 4072 Australia; 9 School of BioSciences, University of Melbourne, Parkville, VIC 3010 Australia

## Abstract

In the coming decades, larger genetic gains in yield will be necessary to meet projected demand, and this must be achieved despite the destabilizing impacts of climate change on crop production. The root systems of crops capture the water and nutrients needed to support crop growth, and improved root systems tailored to the challenges of specific agricultural environments could improve climate resiliency. Each component of root initiation, growth and development is controlled genetically and responds to the environment, which translates to a complex quantitative system to navigate for the breeder, but also a world of opportunity given the right tools. In this review, we argue that it is important to know more about the ‘hidden half’ of crop plants and hypothesize that crop improvement could be further enhanced using approaches that directly target selection for root system architecture. To explore these issues, we focus predominantly on bread wheat (*Triticum aestivum* L.), a staple crop that plays a major role in underpinning global food security. We review the tools available for root phenotyping under controlled and field conditions and the use of these platforms alongside modern genetics and genomics resources to dissect the genetic architecture controlling the wheat root system. To contextualize these advances for applied wheat breeding, we explore questions surrounding which root system architectures should be selected for, which agricultural environments and genetic trait configurations of breeding populations are these best suited to, and how might direct selection for these root ideotypes be implemented in practice.

## Food security and crop root systems

Wheat, which supplies approximately 20% of human calories and protein, is vitally important for global food security (Tadesse et al. 2019), yet in some years global stocks-to-use ratios have reached critical levels (Bobenrieth et al. 2013). It has been estimated that the current relative rate of genetic gain of 1% per annum is insufficient to match the projected demand of an increasing human population by 2050 (Hickey et al. 2019; Hall and Richards 2013), and this is exacerbated by climate change (Asseng et al. 2015). Thus, there is a pressing need to adopt technologies that can accelerate the rate of genetic gain in important food crops (Lenaerts et al. 2019; Cobb et al. 2019).

Historically, the consistent incremental gains delivered by modern cereal breeding have relied predominantly on direct visual selection for improved performance, and this has almost exclusively been based on assessment of the above-ground parts of the plant. This overlooks direct selection of the root system, due to the practical difficulties in assessing the ‘hidden half’ of crop plants (Bishopp and Lynch 2015). Roots are fundamentally important for the plant, mediating the water and nutrient uptake required for growth, and are a source of chemical and hydraulic signals that modulate shoot growth and physiology. In many published studies on crop root systems, it is common to find the suggestion that certain root traits should be selected in breeding programmes, but without further note on how that would happen. In contrast, there is a popular notion that breeding for optimizing root systems of crop species is too difficult and/or expensive. Recent research is showing that breeding for improved root systems is increasingly feasible, with development of tangible tools such as high-throughput phenotyping systems and molecular markers that pre-breeders and breeders can use in practice (Munns et al. 2012; Wasson et al. 2020). Nevertheless, there are few published reports of released cultivars grown by farmers that have been developed with specific selections for root traits (excepting root crop species). One notable example is the upland rice cultivar PY84, which was developed using marker-assisted selection for four quantitative trait loci (QTL) controlling deeper rooting and improved drought tolerance, and which is now grown by farmers in Eastern India (Steele et al. 2013; Steele (personal communication)).

**Fig. 1:**
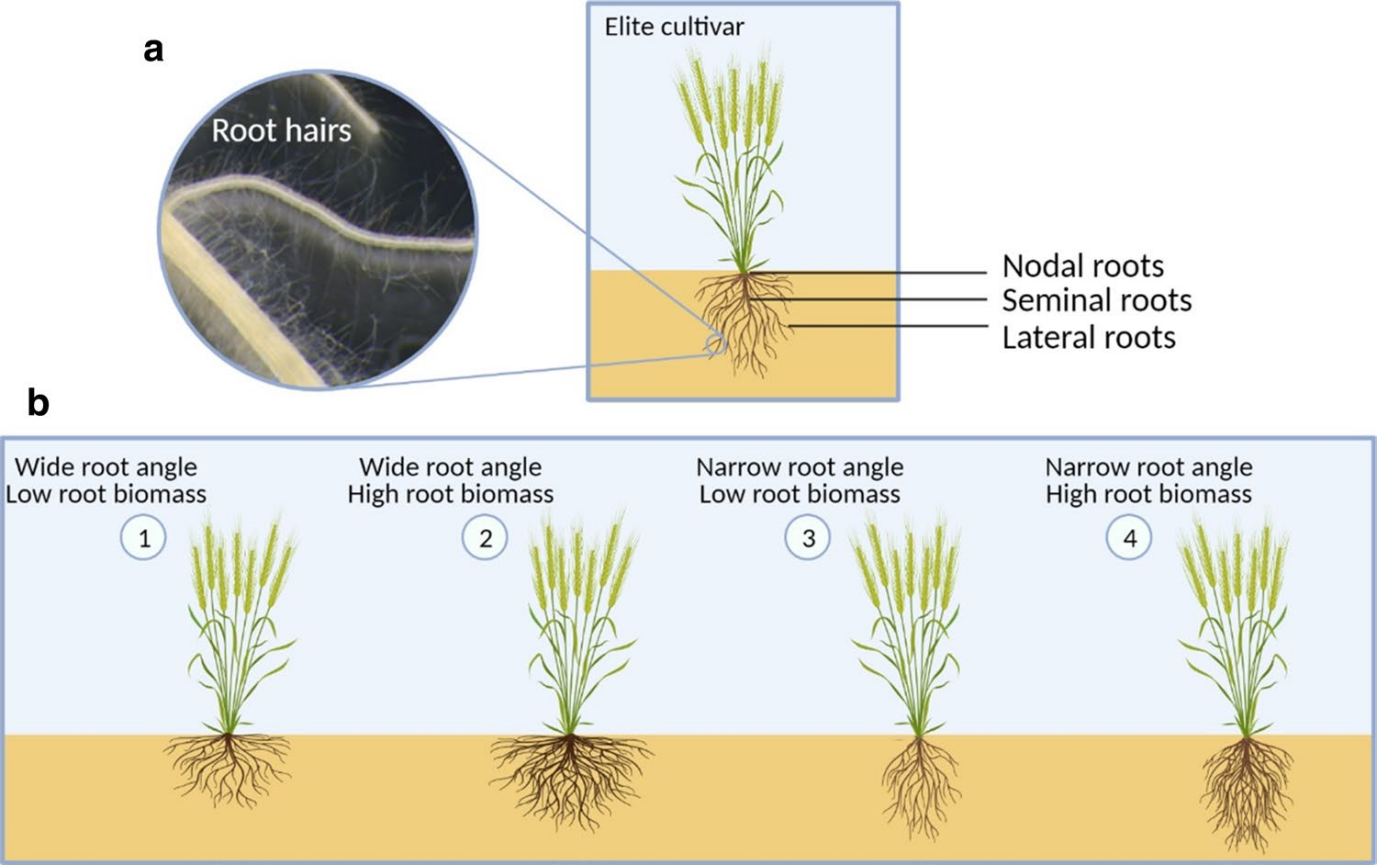
Diagram illustrating root system architectures of wheat. **a** Principal features of the wheat root architecture. **b** An example of different root ideotypes that can be generated by combining different genetic loci controlling root angle and root biomass. Image was created with Biorender.com

Validation of the phenotypes targeted by crop breeding programmes within real-life production environments is essential (Passioura 2012), and for obvious reasons it is particularly challenging to observe roots in situ in the field. However, root system architecture (RSA, the spatial and temporal colonization of roots in the soil; Fig. 1) is clearly important for acquiring soil resources when they are limiting (Lynch 1995). Notable examples are root proliferation in superficial soil layers for P acquisition (Lynch 2019; Henry et al. 2010), placement of roots in deep soil layers to access moisture during drought (Lopes and Reynolds 2010; Hurd 1974; Kirkegaard et al. 2007; Rich et al. 2016) and a strong root plate to minimize lodging (Dreccer et al. 2020; Piñera et al. 2016). However, it is not necessarily obvious what RSA ideotype is needed to achieve the greatest gains in crop yield potential; i.e. the yields obtained under optimal growing conditions (not limited by water, nutrients, light, etc.). Thanks to modern varieties, agricultural practices, machinery and technology, farmers are able to produce outstanding yields under the best conditions. For example, the 2020 world record for wheat grain yield was 17.389 t/ha, grown in New Zealand (https://tinyurl.com/y44xpsgr). We know what that crop looked like above ground, but surprisingly little of what it looked like below ground. Modelling (Lilley and Kirkegaard 2011) and experimental work (Severini et al. 2020) have shown that deeper, more dense root systems are correlated with high yields under optimal growing conditions.

## Climate change and yield ‘resiliency’

Alongside yield potential, yield resilience/stability across a range of environmental conditions is also necessary to minimize seasonal volatility in production and grain quality (Nuttall et al. 2017), especially as growing conditions are becoming less predictable under climate change (Powell et al. 2012; Mickelbart et al. 2015; Scher and Messori 2019). Because crossover behaviour is common, there is no guarantee that a genotype with high yield in the absence of limitations in water or nutrients would also perform well in low input, stressed or marginal environments (Bustos-Korts et al. 2019). Nevertheless, it has been shown that conventional breeding selection has identified high yielding material adapted to poorer conditions by starting with germplasm with good yield potential (Voss-Fels et al. 2019). It is important to understand which RSA works best in which agricultural context, or at least to provide breeders with a range of RSA types to select empirically which is most successful in different target environments, and in the context of different germplasm pools with varying component trait configurations (e.g. Messina et al. 2011). Climate change presents an additional challenge to breeders (Zampieri et al. 2017). In the past, by selecting for yield in each cycle breeders could improve adaptation to local environments, effectively keeping pace with gradual changes in climate. With the current rate of change (and increase in variability) in climate, a key question is whether current breeding practices will continue to be able to deliver the required rate of genetic progress? Perhaps it is too risky to wait to find out, as the development time from initial cross to released wheat variety is approximately 8 years.

At the outset it is valuable to have a clear view of what ‘climate resilience’ means in order to understand what features of a root system would best support a ‘resilient’ crop. In addition to increased air temperatures and incidence of heat stress, climate change models also predict changes in rainfall patterns, with an increase in severity and frequency of drought in some regions, and increased probability of flooding in others (Gornall et al. 2010; Hammer et al. 2020). A ‘resilient’ crop should minimize the potential losses in yield when these conditions prevail. A number of root traits could be important, depending on the environment. During drought, when surface soil layers have been depleted of moisture, root systems that extend deeper into the soil profile may be able to access water (provided that subsoils are replenished by seasonal rainfall), which can help sustain transpiration and photosynthetic rates. In other environments where rainfall is limited and sporadic, the presence of roots in superficial soil layers that remain viable and able to capture water from intermittent rainfall may be advantageous.

The global rise in temperatures may have a smaller direct effect on roots than shoots, as soil temperatures at depth are buffered against fluctuations in air temperatures (Kätterer and Andrén 2009), but some evidence suggests that RSA is sensitive to changes in soil temperature (Luo et al. 2020). Many genes responsive to temperature have pleiotropic effects on both shoot and root traits (e.g. Voss-Fels et al. 2018). Heat stress effects on shoots can be ameliorated in part by maintaining evaporative cooling of the plant through transpiration, and here root systems play an important role (Lopes and Reynolds 2010). During periodic flooding, roots with increased ability to form aerenchyma would confer an advantage, and such ‘cheaper’ roots that have less cortical cell burden per unit length and therefore less metabolic costs can also be beneficial in dry conditions (Chimungu et al. 2019; Klein et al. 2020). Climate change will likely alter the profile of root pest and disease pressures (Juroszek and von Tiedemann 2013), so incorporation of durable sources of resistance represents another form of resilience. A ‘resilient’ variety is one with traits that confer a better likelihood of performing well given the stochastic nature of local weather conditions that define a particular growing environment. We argue that breeders no longer need to consider root systems as a ‘one size fits all’ component and hope for the best. Rather, by introducing RSA ideotypes with specific morpho-physiological traits now into breeding pipelines should improve climate resilience at a faster pace than random occurrence of advantageous RSA. In this review we present the case for tailoring root systems by incorporating more than one component RSA trait at a time (e.g. Fig. 1), outline the tools breeders need to bring about these improvements and provide some examples where progress is happening.

## A tailored root system: the traits that define root architecture and their potential use for improved crop performance

Unlike the ‘taproot’ system of dicotyledonous plants (in which the primary root forms the main root with branching forming secondary, lateral roots and root hairs), monocots such as wheat have root systems comprised of a wide network of finer roots. These consist mostly of nodal roots that arise from the stem nodes, in addition to the seminal roots, lateral roots and root hairs (Hochholdinger et al. 2004) (Fig. 1). Due to research efforts over more than 150 years, we understand quite a lot about how roots grow and develop. From description of root tropisms by Darwin, to field excavations of entire root systems by Weaver in the 1920s (Rich and Watt, 2013), through to cloning genes controlling root development and cell fate (Motte et al. 2019), the published information on roots is extensive. Darwin’s fascination with the root tip was well placed, because the complex signalling that occurs between the root apex and the cells within the root growth zone, plus signals from plant tissues distal to the growth zone, all determines the growth trajectory of the nascent root, and therefore the establishment of the final architecture of the root system fixed in place in the soil. The initiation of root growth through production of root primordia, and the number and spatial arrangement of new root tips are also critical to formation of RSA. Crop plants such as wheat have embryonically formed primary and seminal axile roots and post-embryonically formed, nodal axile roots (for more information on wheat root nomenclature, see Watt et al. 2008). Seminal and nodal axile roots form up to three levels of branch roots, with at least five types of anatomical arrangements. Modification of RSA is strongly affected by root branching (Osmont et al. 2007); branches comprise most of the root length after the four-leaf developmental stage of wheat (Watt et al. 2008). Hydrotropism is the ability of roots to initiate lateral root primordia localized to available water (Bao et al. 2014) and to grow new main roots after a rewetting event (Sebastian et al. 2016). This increases the plant’s plastic response to water shortages and therefore increases resource efficiency by only growing roots where and when they can be useful for water and nutrient uptake (Comas et al. 2013). Hard soils with high penetration resistance present another challenge to roots in many field situations. This can result from mechanical compaction or the increase in soil strength that accompanies soil drying (Whalley et al. 2008). In these problem soils, a genotype with a deep-rooted RSA may not be able to express that phenotype. Therefore, the ability of roots to penetrate hard soils, or more likely, to ‘find’ the cracks, pores and fissures in and between soil peds is an important characteristic of root growth in some environments (Atkinson et al. 2019a, b). It is conceivable that genotypic variation may exist in the oscillatory movement of root tips that is important for this type of soil exploration (Taylor et al. 2020), and perhaps this could be exploited through breeding.

Much of the cereal root system is composed of fine (or lateral) roots and the entire root system is covered with root hairs (Nestler et al. 2016a, b). The rhizosheath, the soil-polysaccharide complex that adheres to roots, relies on mucilage secretion and the presence of root hairs, which enmesh soil particles (Watt et al. 1994; George et al. 2014). Longer root hairs favour larger rhizosheath formation, which also depends on soil water content (Haling et al. 2014) and root type (Nestler et al. 2016a, b). Rhizosheath characteristics may impact plant performance, such as enhancing drought tolerance due to the mucilage water-holding capacity, which attenuates the decline in rhizosphere hydraulic conductivity that occurs during soil drying (Zickenrott et al. 2016).

Compounds released by roots account for 5–40% of total carbon fixed by the plant (Whipps and Lynch 1983; Badri and Vivanco 2009), thus it is likely that root secretions confer a significant benefit, perhaps by allowing plants to tailor the root microbiome to their advantage. There is evidence that rhizosphere microbes can contribute to the uptake of minerals (Bais et al. 2006), tolerance to drought (Kim et al. 2012) and salinity (Zhang et al. 2008; Fatima et al. 2019). Rhizosphere dynamics are complex (McCully 1999) and microbial communities change in response to many factors, including host plant genetics (Aira et al. 2010; Schmidt et al. 2016; Yu and Hochholdinger 2018). Thus, it is conceivable that as we gain more understanding of these factors, varieties can be bred to support a managed microbiome that improves resilience of the crop.

**Fig. 2:**
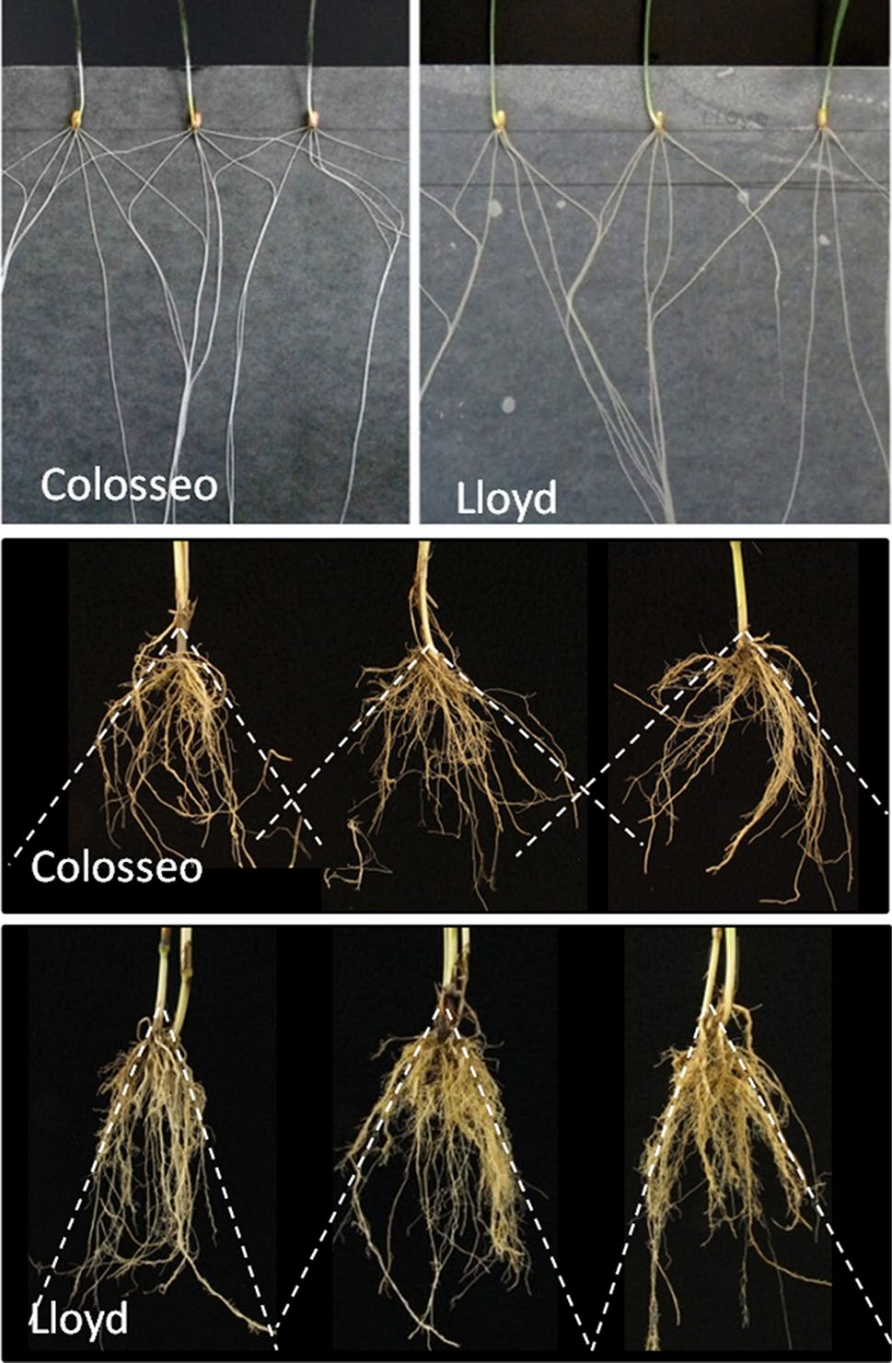
Correlations between seedling stage and adult plant root phenotypes for the founders of a bi-parental durum wheat mapping population. Top panel: Seminal root system grown on germination paper. Middle panel: wide crown root angle in cv. Colosseo from field-excavated plants. Bottom panel: narrow crown root angle in cv. Lloyd from field-excavated plants

The list of root traits highlighted above is by no means comprehensive, and there is a plethora of phenotypes that could confer advantages to crop performance in different growing conditions, many of which have been reviewed elsewhere (e.g. Tracy et al. 2020). Here we consider two broadly defined traits in more detail, due to focus on these phenotypes in recent wheat research: root angle and root biomass. These can be considered ‘coarse’ traits as the phenotype has several layers of expression, so requiring careful definition. To determine root angle, root initials grow along a certain vector in relation to gravity, according to the gravitropic setpoint angle (GSA; Roychoudhry et al. 2017). Growth continues along this trajectory set by the GSA, such that root tissue is established at a fixed angle to vertical. The GSA may change as the root matures or as the root tip responds to local stimuli it encounters in the rhizosphere. Although it is well known that the GSA is set by auxin gradients within the root tip (Toal et al. 2018; Overoorde et al. 2010; Waite et al. 2020), the number of genes and their function in the network regulating this gradient are less well characterized. Two desirable features of root angle at the seedling stage make it particularly suited for genetic studies: the limited effort needed for its measurement in the large numbers of lines that are needed for genetic analysis and its high heritability (Sanguineti et al. 2007; Canè et al. 2014). Notably, a significant correlation has been reported between seminal root angle at the seedling stage and crown root angle measured at maturity in the field (Maccaferri et al. 2016; Alahmad et al. 2019), and as illustrated in Fig. 2. This finding is important because seedling assays are typically conducted on individual plants grown in isolation from other plants, whereas in the field, individuals are members of a population where roots interact with those from neighbouring plants (and sometimes weeds). Indeed, spacings between plants within and between rows can affect RSA (Hecht et al. 2019). Roots with a narrow GSA (i.e. where root tips respond strongly to gravity and so grow closer to vertical) generally result in an overall root system that places more root biomass in deeper soil layers, compared with a root system with a wider GSA that has a greater distribution in the upper soil layers (Alahmad et al. 2019; Lynch 2013; Oyanagi et al. 1993). A deeper root system allows greater access to soil moisture during water deficit, at least in environments that allow replenishment of water in deeper soil layers by seasonal rainfall. Plants with more root biomass at depth could be better at capturing soluble nitrogen before it leaches into groundwater. For example, studies of wheat lines with the chromosome 1RS.1BL translocation from rye show the introgression is associated with greater root biomass at depth and smaller nitrate concentration in leachate than the parent line that lacks the translocation event (Ehdaie et al. 2010). Yield advantages for deeper roots in the field have been demonstrated in bread wheat (Lopes and Reynolds 2010; Li et al. 2019a, b) and durum wheat (Maccaferri et al. 2016; El Hassouni et al. 2018), as well as in rice (Uga et al. 2013), barley (Robinson et al. 2018), triticale (Severini et al. 2020), maize (Gao et al. 2016; van Oosterom et al. 2016) and sorghum (Mace et al. 2012). The fundamental question of whether there is a link between root angle and depth under field conditions has not yet been answered for wheat. Additionally, more complex questions remain, such as how root angle and root biomass interact to promote water acquisition under different soil water availability scenarios, how root biomass and angle relate to above-ground plant architecture in terms of biomass partitioning, and how root traits interact with other above-ground physiological traits.

## Phenotyping methods for characterization and exploitation of root system architecture

To improve a trait in new varieties, including root systems, breeders typically need three fundamental inputs: (1) donor germplasm that contain favourable alleles for the target trait, (2) a way to identify the trait, either by appearance (phenotypic selection) or by the presence of a particular allele (marker-assisted selection) and (3) the human and capital resources to implement the process of selection and breeding. The decision on how much resource to commit to a trait partly depends on whether the value it brings to the final product pays for the effort (and compensates any yield penalties that may come with it). Therefore, the tools and the evidence need to be in place to allow breeders to get such improvements into the hands of farmers.

To best explore the opportunities for the targeted design and selection for RSA traits, appropriate phenotyping methods must be used. Given the complex nature of roots and the difficulties in accessing them when grown in soil, the choice of phenotyping methodology should consider both the root trait to be targeted, and the growth system under which the roots are to be assessed. For example, phenotyping root systems of young plants has practical advantages, as plants can be screened under controlled environment conditions for short periods of time, from two days to a few weeks, so allowing larger numbers of plants to be screened. While controlled conditions typically lead to more reproducible phenotypic results (Poorter et al. 2016), seedling root growth does not always correlate well with mature plants or plants grown in the field (e.g. Bai et al. 2019). The strength of young plant traits as predictors of traits in mature plants depends on many factors, such as the underlying genetic factors, genetic background of the test materials, phenotyping methods and nature of the test environments. Therefore, compromises between complexity of the experimental system and the reliability and reproducibility of the resulting phenotypes must be found. In a breeding context, the choice of the phenotyping strategy would also be affected by the stage of the breeding programme (e.g. line development versus yield testing phase). Keeping these considerations in mind, here we give a brief summary of the methods commonly used to investigate RSA.

### Small plant approaches: from filter paper and agar to rhizotrons

**Fig. 3:**
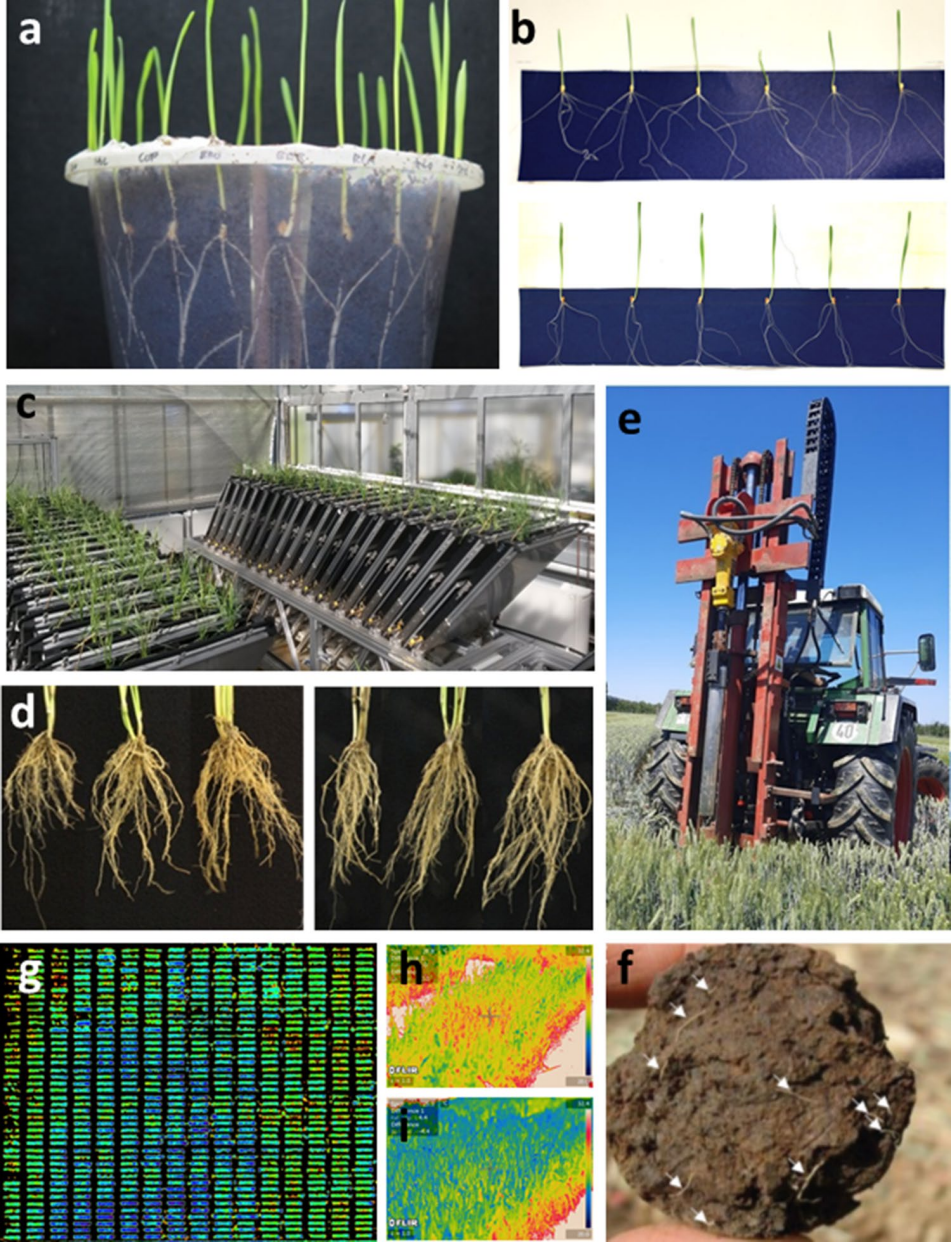
Examples of wheat root phenotyping methods. **a** The ‘clear pot’ seedling method (Richard et al. 2015). **b** Germination paper method, with the upper and lower panels illustrating seedlings with wide and narrow seminal root angle, respectively. **c** Rhizotrons at the Forschungszentrum Jülich. **d** Root crowns excavated from the field (e.g. York et al. 2018). **e** Field-based soil coring via a tractor-mounted rig, with **f** a view of a broken soil core allowing roots to be counted (arrows) (from Wasson et al. 2016). **g** A false-colour orthomosaic thermal image of a field trial of a wheat mapping population (HiBAP2Y18_2H), planted at the CIMMYT field station in Obregón, Mexico. Images were captured using a thermal camera mounted on a UAV (Zenmuse XT, DJI). Cooler canopy temperatures are blue, and warmer temperatures are red. Averaging pure pixel values produces a numerical value for canopy temperature for each genotype and plot (0.8 m × 4 m). Previous studies have shown that canopy temperatures are related to transpiration rates and soil water extraction by the root system (see text). The image also shows spatial variation across the trial site and variation within the plot, which have to be taken into account to increase the heritability of the temperature trait values. Close-up images of plots made with a hand-held thermal camera taken within a few minutes of each other, showing contrasts between genotypes in neighbouring plots within the same trial in the UK: **h** cv. Apache, **i** cv. Paragon (color figure online)

Non-invasive, time-resolved observations of roots are possible when growing seedlings in transparent pots (e.g. Richard et al. 2015) (Fig. 3a), on germination paper (e.g. Gioia et al. 2017) (Fig. 3b), agar plates (Nagel et al. 2020), or in paper or cloth pouches (Shorinola et al. 2019; Chen et al. 2020). While some of these growing platforms require sterile conditions and manifestation of the desired trait within two to three weeks, they enable relatively high throughputs of several hundred plants at a time. Alternatively, plants can be efficiently screened for RSA traits in soil-filled glass-walled ‘rhizotrons’ (Fig. 3c), where root growth can be monitored over time (e.g. Nagel et al. 2012; Jeudy et al. 2016). Using rhizotrons to investigate young plants before their roots reach the constraints of the containers (Passioura 2006; Sinclair et al. 2017) allows tracking of natural root system growth in (2D) space and time, or mapping more mature root systems in 3D (Topp et al. 2013). In large rhizotrons, it is possible to observe and measure roots to 4 m depth, but these are not suitable for comparing large numbers of genotypes (Svane et al. 2019). In general, rhizotrons require more space than the paper or agar methodologies described above. However, medium-to-high-throughput rhizotron facilities are currently available at the Forschungszentrum Jülich and Leibniz Institute-IPK, capable of screening hundreds of wheat plants in a single experiment (Fig. 3c).

### Field-based root phenotyping

Root phenotyping in the field is often considered more complex, time-consuming and labour-intensive than seedling approaches. In the method commonly termed ‘shovelomics’, large numbers of root crowns are excavated either using a shovel or manually, depending on the soil conditions (Fig. 3d). This approach is surprisingly fast, with throughput of up to ~ 50 shovel excavations/h/person. However, it is an invasive method and it only allows access to roots growing in the top 20 cm of soil. Nevertheless, when combined with image analysis, shovelomics can provide useful insights into traits such as nodal root number or growth angle of field-grown crops, including wheat (Maccaferri et al. 2016; York et al. 2018; Fradgley et al. 2020). A non-destructive technique for observing field-grown roots over time is also possible by employing ‘minirhizotrons’—transparent cylinders that are placed into a field in a desired position before planting, either with an installed camera for continuous imaging, or with an opening to insert e.g. an endoscope for root imaging at a specific time point. Minirhizotrons give insights into root number and length over time, but they are limited to pre-defined positions (Crocker et al. 2003). Assessing root structure in the field at greater soil depths can be undertaken using soil coring, typically using a tractor-mounted hydraulic system (Wasson et al. 2016), followed by core breaking (Fig. 3e, f), washing and root measurement. A new approach to the phenotyping of soil cores involves core breaking and counting of roots at the core face without washing. Soil coring is more time intensive (< 5 cores/h/person) and costly than shovelomics, requiring specialized equipment (Wasson et al. 2016). However, the data obtained are roots per soil volume, potentially to a depth of 2 m, making them useful for crop growth models that require a root length density at each soil layer. To increase throughput, Wasson et al. (2016) recently developed a method for automated detection of roots at the core break interface via image analysis and UV (365 nm) LED illumination that increases the contrast between the roots and the soil.

### Remote sensing for indirect root phenotyping

The throughput of direct observation of root systems in the field is currently insufficient for routine application in breeding pipelines. An alternative approach is to use remote sensing methods to phenotype above-ground traits as a proxy for below-ground traits. Remote sensing has been used to estimate soil water availability, relative root depth and root/shoot ratio at the genotype level. For example, a direct phenotypic and genetic link has been found between wheat root mass and canopy temperature under both drought and hot/irrigated conditions (Lopes and Reynolds 2010; Pinto et al. 2010). Subsequent root excavation work showed that the lines with cooler canopies (and the associated genetic loci associated with this trait) express a larger root mass under both drought and heat, though with different root distribution profiles, reflecting where water was available to roots under the distinct environments (Pinto and Reynolds 2015; Li et al. 2019a, b). Parallel work investigating the spectral reflectance ‘water index’ showed its sensitivity to genotypic effects on both leaf and soil water potential in drying soils (Gutierrez et al. 2010), the latter being a direct function of water uptake by roots. Since canopy evapotranspiration is a function of root access to water and the demand created by the leaf canopy and atmosphere energetics, canopy temperature and water index signals likely predict relative root capacity (i.e. root/shoot ratio) rather than root mass per se. Normally, canopy temperature is predominantly driven by evaporative cooling of the leaf surface via transpiration, which is a function of feedback control loops between stomatal conductance and plant hydraulic conductivity, controlled in part by root vascular capacity. However, as the soil dries or evaporative demand increases, canopy temperature is increasingly determined by the capacity of root systems to supply water for transpiration. Thus, canopy temperature and water index signals become progressively more sensitive to genetic differences in root capacity as soil water becomes limiting. The development of such a root index (based on modelling of remote sensing data under well-defined environmental conditions) would have profound implications for our ability to rapidly screen root capacity at a breeding scale. In large field trials, spatial and temporal variation in canopy temperatures can be minimized via rapid imaging using thermal cameras mounted on drones or piloted aircraft (Deery et al. 2019; Fig. 3g).

Additionally, non-invasive methods based on geophysical techniques are being developed, such as ground penetrating radar (GPR). GPR uses high-frequency radio waves to detect roots by exploiting differences in electromagnetic properties between roots and the soil and has been used to estimate growth of storage roots in cassava (Delgado et al. 2017). Though typically more successful when applied to larger (> 1 cm diameter) roots, GPR has been shown to predict bulk root biomass and diameter in winter wheat (Liu et al. 2018). Finally, living root biomass distribution can be estimated by indirect measures of root activity, such as water uptake. Wheat genotypic differences in water uptake in deep soil layers have been demonstrated using a frequency domain reflectometry type soil moisture sensor (Ober et al. 2014), although this method also requires installation of soil access tubes in the field. Electrical resistance tomography (ERT) and electromagnetic inductance (EMI), which are commonly used to map soils at different depths, have been used to quantify changes in apparent conductivity related to soil moisture content, and thus to discriminate soil drying profiles of different genotypes in the field (Whalley et al. 2017).

### Emerging developments in root phenotyping

Among the emerging trends in root phenotyping are improvements in non-destructive methods for controlled environments (Atkinson et al. 2019a, b) and direct, deep phenotyping under field conditions (Wasson et al. 2020). Contemporary root phenotyping platforms under controlled conditions now routinely co-image both shoots and roots, greatly speeding up the introgression of desirable shoot and root phenotypes (Tracy et al. 2020). Although they require specialist equipment and facilities, magnetic resonance imaging and micro-X-ray computed tomography allow non-destructive, time series 3D imaging of RSA and rhizosphere properties in fine detail (Mairhofer et al. 2012). Improvements in scan times and use of robotics has increased sample throughput (Tracy et al. 2020). For selection of root anatomical traits at the cellular level such as aerenchyma, laser ablation tomography has increased throughput compared with conventional microscopic techniques (Galindo-Castañeda et al. 2018).

## The genetics and genes controlling RSA

Wheat, along with the related grass crop species rice and maize, represent the three most important staple crops globally. Our current understanding of the genes controlling root traits in wheat is relatively sparse. Therefore, here we first give a brief overview of the current knowledge in maize and the model grass species, rice.

### Model roots: cloned RSA genes in rice and maize

At least eight maize genes controlling root system architecture have been identified to date (recently reviewed by Hochholdinger et al. 2018). Of these, key elements of seminal, shoot-borne and lateral root development are controlled by genes in the auxin signalling pathway: *rootless with undetectable meristem1* (*rum1*), *rtcs*, *rtcs-like* (von Behrens et al. 2011; Taramino et al. 2007; Xu et al. 2015). In contrast, genes controlling root hair formation have been found to regulate the properties or synthesis of cellulose and the cell wall (*roothairless3* (*rth3*), *rth5* and *rth6*) (Hochholdinger et al. 2008; Nestler et al. 2014; Li et al. 2016) or exocytotic vesicle docking (*rth1*, *big embryo1*) (Wen et al. 2005; Suzuki et al. 2015). Notably, two major QTL controlling the number of seminal roots in maize co-map with the root developmental genes *rtcs* and *rum1* (Salvi et al. 2016), hence supporting the hypothesis that mutants and QTL are underpinned by the same loci but with much stronger additive effect in the former compared with the latter (Robertson 1985; Bohn et al. 2006). Genes controlling RSA have also begun to be characterized in rice (reviewed by Meng et al. 2019), with two root trait QTL having been map-based cloned to date. The first was the genetic locus *DEEPER ROOTING 1 (DRO1)*, found to be encoded by a protein with two putative N-myristoylation sites associated with lipid modification (Uga et al. 2013). *DRO1* influences seedling root angle via modulating gravitropic response, with the deep rooting allele found to improve grain yield under field drought stress (Uga et al. 2013; Arai-Sanoh et al. 2014). More recently, the rice QTL *qRT9* controlling root thickness and length was found to encode the basic helix-loop-helix transcription factor, *OsbHLH120* (Li et al. 2015). *OsbHLH120* expression was induced by application of various conditions in hydroponic culture (NaCl, polyethylene glycol and abscisic acid); the observation that this response was stronger in plants carrying the long/thick root allele indicates that selection for this allele provides an opportunity for breeding drought tolerant lowland rice varieties.

### Genetic control of RSA in wheat

In wheat, little is known about the underlying genes controlling root architecture. Aside from the complexities in root phenotyping, this is likely due to the large, highly repetitive and polyploid nature of the wheat genome (17 Gbp for hexaploid bread wheat, approximately 40 times that of rice) as well as the buffering effects of homoeologous genes present within its three sub-genomes (Borrill et al. 2019). Indeed, although many hundreds of RSA QTL have been identified (e.g. as summarized in the meta-analysis conducted by Soriano and Alvaro 2019), only two genes controlling RSA in wheat have been formally identified. The first is *VERNALIZATION 1 (VRN1)*, which influences root angle at all stages of plant growth (Voss-Fels et al. 2018). Interestingly, natural variation at *VRN1* has long been recognized as a major-effect gene controlling the transition from vegetative to reproductive growth in wheat (Yan et al. 2003; Cockram et al. 2007). That such a well-studied gene has only recently been shown to be involved in root system architecture perhaps highlights the comparative lack of research resources wheat root traits have previously been afforded. More recently, the barley root angle mutant *enhanced gravitropism 2* (*egt2*) encoding a STERILE ALPHA MOTIF domain-containing protein has been cloned, which results in narrower and lateral root growth angle compared to wild-type, combined with higher response to gravity in an auxin-independent manner (Kirschner et al. 2021). Combining mutations in the A and B genome copies of the orthologous gene in tetraploid wheat resulted in narrower seminal root growth angle, indicating *EGT2* could be a target for root-based cereal crop improvement (Kirschner et al. 2021).

Novel approaches for wheat speed breeding (Watson et al. 2018) and root phenotyping are now converging with the coming of age of wheat genomics resources (reviewed by Adamski et al. 2020). Applied together this can accelerate generation of novel germplasm to test RSA ideotypes in the field. The publication of the fully annotated reference wheat genome assembly in 2018 (IWGSC 2018) has been rapidly followed by the release of 15 additional assemblies of modern bread wheat cultivars (Walkowiak et al. 2020). Together with gene expression databases (Ramírez-González et al. 2018), this allows more rapid discovery and characterization of genes controlling RSA in wheat. For example, forward phenotypic identification of induced mutants affecting root traits similar to that initiated in maize in the 1990s (e.g. Wen et al. 1994; Hochholdinger and Felix, 1998) are now being undertaken in wheat, identifying for example seminal root number mutants at the seedling stage (Shorinola et al. 2019). As these artificially mutated wheat germplasm resources have also been sequenced (Krasileva et al. 2017), reverse genetics approaches focusing on wheat orthologues of genes known to affect RSA in other cereals, such as wheat orthologues of *DRO1* (Ashraf et al. 2019), can also be rapidly identified for subsequent characterization. Translational genomics from model plants such as *Arabidopsis*, which exploits the conservation of gene sequence and function across species, has led to the discovery of novel genes in wheat that control primary root growth and morphology, such as *TaARF4* (Wang et al. 2019) and *TaLAMP1* (Shi and Tong 2020). Similarly, the recent development of genotyping platforms (e.g. Allen et al. 2017) that can rapidly type genetic variants throughout the wheat genome is contributing to the availability of increasingly finely mapped wheat RSA QTLs. Indeed, numerous QTL mapping studies have dissected the genetic control of root traits, with the majority of these involving bi-parental populations (e.g. Maccaferri et al. 2016). These advances in genomic resources should facilitate identification of haplotypes or alleles that can be used to alter RSA in wheat in a targeted manner. A notable example is the identification of a major locus controlling > 50% of the phenotypic variance for primary root length on bread wheat chromosome 2B (Ren et al. 2012), with subsequent proteomics investigation implicating the role of transforming growth factor (TGF)-beta receptor-interacting protein-1 (TaTRIP1) in the control of meristem size (He et al. 2014).

Recently, the use of other population types for genetic analysis has become increasingly common in crops (reviewed by Cockram and Mackay 2018). For wheat root traits, these include the use of genome wide association studies (GWAS) on nested association mapping (NAM) populations and association mapping panels consisting of large collections of varieties or accessions. One of the advantages of these techniques is the possibility to evaluate more than two haplotypes at any given locus, thus allowing a wider range of genetic diversity to be explored. A good example of the potential of these approaches is the investigation of a major genetic locus on chromosome 6A of durum wheat controlling root angle. First identified by GWAS of seminal root phenotype in elite germplasm (Sanguineti et al. 2007; Canè et al. 2014), and subsequently validated on the whole root system under field conditions (Maccaferri et al. 2016; Fig. 2), the effect of this QTL on root angle and agronomic performance was further validated in a durum NAM population (Alahmad et al. 2019, 2020) and via GWAS conducted with Ethiopian durum accessions (Alemu et al. 2021). A recent use of GWAS for analysis of root traits in bread wheat is reported by Voss-Fels et al. (2017), who identified two epistatic loci on chromosome 5B controlling root biomass. Interestingly, the high-biomass allele combination at these loci was found to be absent in elite European germplasm.

In addition to the genetic diversity present in elite germplasm, natural variation present in landraces and related cereal species represents a reservoir of potentially novel alleles that is now beginning to be exploited in the context of RSA traits. For example, assessing a near isogenic line (NIL) pair differing for the introgression of a region of chromosome 7A from wild emmer wheat (*T. durum* subsp. *dicoccoides*) into an elite durum wheat cultivar found the introgression to confer higher yield under drought conditions (Merchuk-Ovnat et al. 2016). This was underpinned by a significant and consistent effect of the wild emmer introgression on RSA (Merchuk-Ovnat et al. 2017). Another example of the beneficial effect of chromosomal introgressions on RSA is the chromosome 1RS.1BL translocation from rye (*Secale cereale* L.) into bread wheat, which affects grain yield and canopy water status (Villareal et al. 1991; Howell et al. 2014). The introgression has since been found to affect RSA traits by maintaining root apical meristem activity over longer time periods, resulting in longer axial roots and improved deep-water capture (Ehdaie et al. 2003; Howell et al. 2019). Conversely, the wild-type bread wheat allele reduced the meristematic activity of the seminal root apex 10 days after germination, resulting in reduced seminal root length and lateral root proliferation. The importance of the lateral/axial root meristematic activity balance in wheat root deepening has also been highlighted in another study involving an alien introgression into bread wheat, in which a translocation from the wheat wild relative *Agropyron elongatum* on chromosome 7D caused impaired lateral root proliferation as a consequence of water deficit, resulting in the maintenance of meristematic activity of axial root apexes (Placido et al. 2013). Chromosomally engineered durum wheat *Thinopyrum ponticum* recombinant lines tested in a range of contrasting rain-fed environments showed associated effects between seminal root angle and yield (Kuzmanović et al. 2018). By exploring root gene transcription in lines contrasting for this introgression, as well as RNAi gene silencing in bread wheat, the gene *LATERAL ROOT DENSITY (LRD)* was identified as a repressor of root growth under drought conditions and was proposed to control the deeper rooting phenotype under drought conditions conferred by the *Agropyron* introgression (Placido et al. 2020).

**Fig. 4:**
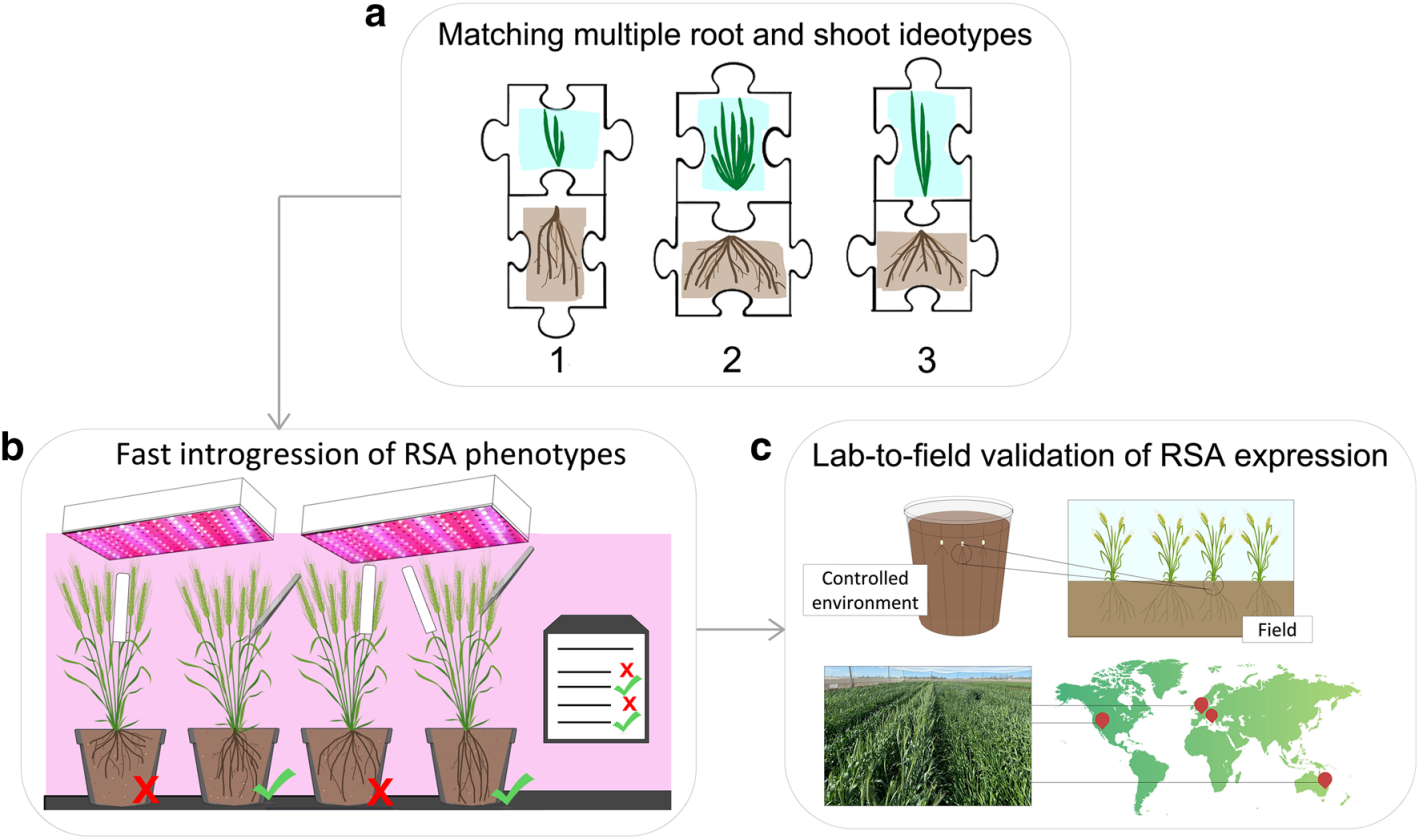
An approach for matching and evaluating multiple root and shoot ideotypes in elite wheat genetic backgrounds adapted to different growing regions. **a** Genetic loci identified as controlling root traits and shoot traits are used as the starting points for **b** back-crossing into elite wheat varieties. ‘Speed-breeding’ growth conditions (Watson et al. 2018), combined with clear-pot root phenotyping (Richard et al. 2015) and marker-assisted selection (Makhoul et al. 2020), allow the development of near isogenic lines (NILs) that combine multiple above- and below-ground traits into elite wheat varieties adapted to different agricultural environments. **c** The targeted root and shoot traits can then be validated in the NIL materials under both controlled and field environments to determine their effects on overall crop performance

Researchers in the pre-breeding community have focused on studying diversity panels and bi-parental mapping populations to discover root trait QTL, but have faced challenges when it comes to precisely quantifying the value of specific root traits. While these populations segregate for root traits, most also segregate for above-ground developmental traits that have a major impact on the timing of water-use and carbon partitioning, for example flowering time and plant height. Therefore, the ability to determine the value of different root traits and their contribution to yield is confounded by above-ground variation. We propose that development of elite introgression lines for target root traits with similar above-ground behaviour would provide valuable materials for evaluating the value of the new trait in different environments or production scenarios (Fig. 4). We further propose that, given the availability of new genomics tools, advances in crop models/genetic simulations, low-cost and efficient phenotypic screening systems and speed breeding technology, this targeted approach to validate root trait QTLs and trait value could help accelerate progress in wheat.

### Genetic engineering and gene editing for precise modulation of RSA in elite wheat germplasm

There are currently few examples of RSA trait introduction into wheat through genetic engineering, due predominantly to the relatively recent establishment of the necessary methodologies and underpinning genomic resources in wheat. Examples include increased wheat root hair development mediated by *TaRSL4-A* expression, as well as expression of the flowering regulator, *VRN1* used to confirm the role of this gene on modulating RSA in both wheat and barley (Han et al. 2015; Voss-Fels et al. 2018). Recent improvements in wheat transformation methods (Debernardi et al. 2020) and the development of new breeding technologies utilizing site-specific nucleases such as zinc finger nucleases, TALENS and CRISPR (Zhu et al. 2020), meaning that precise modification of DNA sequences in elite wheat varieties is now possible. Individual genes, or gene families can be precisely targeted by the nuclease to abolish gene function (Gil-Humanes et al. 2017; Li et al. 2018). The use of ribonucleoprotein complexes to deliver editing components removes the need for foreign DNA to be stably integrated and segregated away so that GM regulation may not be required (Liang et al. 2017). Coupled with availability of a donor template, the less efficient homology-directed recombination pathway can precisely introduce new alleles, creating a desired haplotype without the need for an extensive crossing programme (Shi et al. 2017). As genome editing methodologies have rapidly advanced, modification of the temporal and spatial expression of target genes has now become possible (Lee et al. 2019, Gallego‐Bartolomé 2020). These modifications could have profound implications for the future validation and exploitation of genes underlying RSA QTL without associated linkage drag from closely linked alleles. Recent examples include CRISPR editing of the cotton *GhARG* gene which resulted in increased lateral root formation and, in barley, knockout mutation of the *HvCKX1* gene resulted in greater root length and increased surface area (Wang et al. 2017; Gasparis et al. 2019). While no known RSA genes have been modified in wheat to date, and the generation of full gene-edited knockouts in polyploid crops has proven challenging, recent strategies to improve editing in hexaploid wheat make this a realistic prospect in the near future (Milner et al. 2020). Several countries currently regulate these gene-edited crops as genetically modified events—even if the foreign T-DNA has been removed, leaving just the gene edit. However, the use of ribonucleoprotein complexes or haploid induction systems (Kelliher et al. 2019) to deliver the editing components removes the need for foreign DNA to be stably integrated and segregated away. This approach has been demonstrated in wheat, opening the door for gene-edited wheat that falls outside the scope of current GM regulation (Liang et al. 2017).

## The missing piece of the puzzle: root plasticity

RSA traits are governed not only by genetics but also by environmental conditions, such as soil structure, moisture, nutrient availability, temperature and biotic factors. Quantitative traits such as root angle are controlled by multiple genes, and a systems approach is required to understand how that gene network is regulated, including those elements that respond to environmental signals (Rellán-Álvarez et al. 2016). Thus, within the root system of a plant, or a specific component of that root system, multiple states are possible depending on the local transcriptional, translational and post-translational status of each gene and its interaction within the network, resulting in a plastic expression of the phenotype (which perhaps can be more accurately viewed as an environment-specific trait response). For example, Schneider et al. (2020) showed that in maize, the genes controlling root anatomical traits under different water regimes or locations could be differentiated from sets of genes that were associated with the plasticity of the traits (the proportional change in expression across environments). It remains unclear what aspects of plasticity are maladaptive, neutral or beneficial for productivity. For those traits that show plasticity such as root angle, the targets for breeding would include not only those major-effect genes that establish the setpoint angle, but also those genes that modify the setpoint in response to an environmental signal such as water deficit (Schneider and Lynch 2020). Dynamic developmental root traits such as root growth rate, which must be determined from measurements at more than one time point, may also be important features of plastic responses. Exploiting genetic variability for the regulation of root growth rate (Li et al. 2019a, b) may be one route to increased climate resiliency. For certain target environments, breeders may opt for less plasticity via a combination of alleles that maintain a network status that shows low responsiveness to environmental signals; e.g. consistent maintenance of narrow root angle for conditions where resources are low in the upper soil layers. In other situations, greater plasticity may confer yield advantages, which would be desirable despite low heritabilities for those traits. Such decisions will need to be based on extensive experimental data on genotype, root phenotype and yield and a greater understanding of how those gene networks function.

## Outlook for breeding better wheat root systems

We view the critical steps to underpin future direct selection of root traits for improved crop performance as being: (1) establishment and routine use of high-throughput, high-precision, field-based/field-relevant phenotyping approaches, (2) development of molecular tools with which to track and combine beneficial alleles controlling RSA, and perhaps most critically, (3) detailed knowledge of which RSA are best suited to a given agricultural environment and to the wider genetic architecture of the germplasm used in any specific breeding programme. We expect the coming years will most likely firstly see the molecular characterization of increasing numbers of wheat RSA genes, providing the research community with multiple entry points into the genetic networks that underlie wheat RSA, as well as rapid approaches with which to integrate this knowledge into the targeted design of RSA ideotypes. These include advanced modelling and simulation capabilities to reveal promising RSA-component trait configurations that can be targeted in breeding programmes. Once available, such resources will allow the assessment of specific RSA ideotypes across multiple environments, providing the knowledge base from which these can be most confidently incorporated into future breeding programmes. Over this period, we believe that the ideal of high-precision in-field root phenotyping will most likely be tackled via the continued development of approaches that overlay multiple layers of phenotypic data from direct and indirect measurements of root performance, combined with high-power computing and AI approaches. Of course, these idealized outcomes are composed of a continuum of constitutive advances in research and innovation, and the steps along the way will offer opportunities for immediate practical use for crop breeders. For example, Richard et al. (2018) have recently demonstrated the potential of direct selection in early segregating generations by selecting individual plants contrasting for seminal root angle, which resulted in a significant shift in both phenotype and allele frequency in derived populations. Importantly, the critical steps listed above come together as phenotypic datasets that could be used to enrich functional-structural models that drive RSA computer simulations (reviewed by Tracy et al. 2020). Advances in functional genomics, system biology, modelling and computing power will allow for a more comprehensive analysis of genotypic and phenotypic information to predict the outcome of different allelic combinations for RSA. As the accuracy of these stochastic models increases at the field and farming system level, breeders will be able to run thousands of potential simulated gene combinations before the chosen crosses are made and planted out for field evaluation.

To help support the overall goals outlined here, we have established an international project between researchers and breeders in the UK, Germany, Italy, Australia and Mexico that begins to investigate the three key factors we list above. Following the principles outlined in this review, our aim is to exploit the current advances in RSA gene characterization made in wheat within the consortium and the wider community and translate this into knowledge and wheat germplasm resources adapted to multiple growing environments and countries. In ongoing work, consortium members have created a panel of 80 wheat NILs comprising the four seedling RSA ideotypes depicted in Fig. 1 in four elite backgrounds, in order to test relationships to mature plant RSA and yield under field conditions in different environments. As wheat is a global commodity underpinning food security across the world, we advocate such approaches for the efficient advancement of our understanding of promising and workable below- and above-ground trait configurations, thus helping their efficient exploitation in crop breeding programmes around the world.

## Abbreviations

ERT: Electrical resistance tomography
EMI: Electromagnetic inductance
GSA: Gravitropic setpoint angle
GWAS: Genome-wide association study
KASP: Kompetitive allele-specific PCR
NIL: Near-isogenic line
QTL: Quantitative trait locus
RSA: Root system architecture
SNP: Single nucleotide polymorphism
